# The fecal microbiome and metabolome differs between dogs fed Bones and Raw Food (BARF) diets and dogs fed commercial diets

**DOI:** 10.1371/journal.pone.0201279

**Published:** 2018-08-15

**Authors:** Milena Schmidt, Stefan Unterer, Jan S. Suchodolski, Julia B. Honneffer, Blake C. Guard, Jonathan A. Lidbury, Jörg M. Steiner, Julia Fritz, Petra Kölle

**Affiliations:** 1 Clinic of Small Animal Medicine, LMU University of Munich, Munich, Germany; 2 Gastrointestinal Laboratory, Department of Small Animal Clinical Sciences, College of Veterinary Medicine and Biomedical Sciences, Texas A&M University, College Station, Texas, United States of America; 3 Napfcheck, small animal nutrition consultation, Munich, Germany; University of Illinois, UNITED STATES

## Abstract

**Introduction:**

Feeding a Bones and Raw Food (BARF) diet has become an increasing trend in canine nutrition. Bones and Raw Food diets contain a high amount of animal components like meat, offal, and raw meaty bones, combined with comparatively small amounts of plant ingredients like vegetables and fruits as well as different sorts of oil and supplements. While many studies have focused on transmission of pathogens via contaminated meat and on nutritional imbalances, only few studies have evaluated the effect of BARF diets on the fecal microbiome and metabolome. The aim of the study was to investigate differences in the fecal microbiome and the metabolome between dogs on a BARF diet and dogs on a commercial diet (canned and dry dog food).

**Methods:**

Naturally passed fecal samples were obtained from 27 BARF and 19 commercially fed dogs. Differences in crude protein, fat, fiber, and NFE (Nitrogen-Free Extract) between diets were calculated with a scientific nutrient database. The fecal microbiota was analyzed by 16S rRNA gene sequencing and quantitative PCR assays. The fecal metabolome was analyzed in 10 BARF and 9 commercially fed dogs via untargeted metabolomics approach.

**Results:**

Dogs in the BARF group were fed a significantly higher amount of protein and fat and significantly lower amount of NFE and fiber. There was no significant difference in alpha-diversity measures between diet groups. Analysis of similarity (ANOSIM) revealed a significant difference in beta-diversity (p < 0.01) between both groups. Linear discriminant analysis effect size (LefSe) showed a higher abundance of *Lactobacillales*, *Enterobacteriaceae*, *Fusobacterium* and, *Clostridium* in the BARF group while conventionally fed dogs had a higher abundance of *Clostridiaceae*, *Erysipelotrichaceae*, *Ruminococcaceae*, and *Lachnospiraceae*. The qPCR assays revealed significantly higher abundance of Escherichia coli (*E*. *coli)* and *Clostridium (C*.*)*. *perfringens* and an increased Dysbiosis Index in the BARF group. Principal component analysis (PCA) plots of metabolomics data showed clustering between diet groups. Random forest analysis showed differences in the abundance of various components, including increased 4-hydroxybutryric acid (GBH) and 4-aminobutyric acid (GABA) in the BARF group. Based on univariate statistics, several metabolites were significantly different between diet groups, but lost significance after adjusting for multiple comparison. No differences were found in fecal bile acid concentrations, but the BARF group had a higher fecal concentration of cholesterol in their feces compared to conventionally fed dogs.

**Conclusion:**

Microbial communities and metabolome vary significantly between BARF and commercially fed dogs.

## Introduction

Bones and Raw Food (BARF) has been gaining popularity among dog owners in the last years. These diets try to imitate the feeding behavior of the wolf. Consequently, BARF diets contain raw meat, different offal and raw meaty bones, representing the wolf’s prey (70–80% of the diet) [1, 2], which are mostly combined with vegetables and fruits to represent the digestive tract content of the prey animals (remaining 20–30% of the diet). Not being an integral component of the natural food spectrum of the wolf, carbohydrates are often eliminated from the diet or only used in small amounts. To optimize nutrient supply, many dog owners add oils and different dietary supplements like eggshells, seaweed meal, and herbs. Nevertheless, nutritional imbalances are common in BARF diets [3].

It is well known that the fecal microbiome is affected by several factors. Previous studies have shown that the health status is one of the strongest factors shaping the composition and diversity of the fecal microbiome. For example, in dogs suffering from exocrine pancreatic insufficiency (EPI) [4] or inflammatory bowel disease (IBD) [5], the microbiota structure is different and diversity is reduced when compared to healthy dogs. Another important factor that influences gut microbiome is the composition of the diet, especially the impact of dietary fiber on microorganisms [6, 7]. Apart from fiber, different amounts of protein and carbohydrates can also affect the composition of fecal microorganisms [8], but it is important to note that it needs an appropriate compositional change of the diet to achieve significant changes in the microbiome [9, 10]. Only a limited number of detailed studies has evaluated the effect of feeding BARF diets on the fecal microbiome and metabolome of healthy dogs [11, 12]. Thus, the aim of the study was to evaluate differences in the microbiome and functional metabolites between client owned dogs on a BARF vs. a commercial diet.

## Methods

### Animals

Owners verbally gave an informed consent to participate in the study and completed a questionnaire that contained questions about patient`s characteristics and feeding habits (S2 and S3 Files). An approval by an ethics commission in Bavaria is only required if the study may be associated with possible pain or suffering of the patients due to study procedures. As this was a study not involving any animals directly, but exclusively using historical information and gathering of feces after defecation, such an approval was not necessary and thus not sought.

A total of 46 dogs without history of gastrointestinal (GI) problems on the current diet were included. The BARF group consisted of 27 dogs from 20 different owners, 15 being female and 12 male. The youngest dog was seven months, the oldest 15 years (median 4 years). The dogs were recruited through calls for participation via homepage and staff members of the Small Animal Medicine Clinic. Some of the participating dog owners were members of different BARF-forums. They were allowed to share the link of the small animal clinic homepage with further information about the study in their discussion forums if it was allowed to post these kinds of contents.

Within the BARF group, three dogs had musculoskeletal problems, two dogs had an eye disease, two dogs suffered from cardiac diseases, one from skin disease, and one dog was diagnosed with epilepsy. As all of the dogs with history of an illness showed a good general health status and had a normal stool quality (Purina fecal score 2–3), these dogs were kept in the trial. Two dogs received antibiotics during the last 3–5 months, six dogs in the last 6–12 months, 16 dogs were not treated with antibiotics for at least 12 months and two dogs had never received antibiotics. One dog was purchased four weeks ago without detailed information of medical treatment available.

As a control group, 19 dogs from 17 owners were recruited from clinic staff and acquaintances. Ten dogs were female and nine male, with a median age of 5 years (range 2–15 years). Fifteen dogs had no history of any disease, three dogs had a food intolerance with no symptoms on the current diet and one dog had suffered from a pyelonephritis a short time ago, treated with antibiotics four weeks prior to fecal sampling. Another dog received antibiotics because of an accident 16 days prior to sample collection. Both dogs were kept in the trail because statistical results were not different compared to the rest of the dogs in the control group. Only one of the other dogs received antibiotics 9 months ago, whereas 14 dogs were not treated with antibiotics for at least 12 months and two dogs had never received antibiotics.

### Diets

All but one owner answered the questions about the BARF diet composition of their dogs. This dog was excluded in the precise calculation of the BARF diet but kept in the trial because the owner fed a typical BARF diet depending on a high amount of animal components and no addition of carbohydrates. Two dogs were fed a BARF diet for four weeks, two dogs for two or four months respectively, the rest of the dogs were fed a BARF diet for at least six months (0.6 years up to 9 years).

Owners of BARF fed dogs used various diet compositions including raw meat (e.g. beef, chicken, lamb, horse, goat) and different offals (e.g., rumen, omasum, heart, lungs from different types of animals). Twenty-one of these dogs also received raw meaty bones. While some owners bought these products at supermarkets, butchers or directly from hunters, other owners bought the dog`s meat at specialized BARF-(online)-shops. For most dogs, these animal-based products were combined with vegetables, different kinds of oil, and sometimes eggs and dairy products. Owners of twelve dogs did not feed any carbohydrates in their dogs’ main diet.

Thirteen dogs of the control diet received dry dog food, three dogs canned dog food and three dogs a combination thereof. One of the dog owners who fed a combination of wet and dry food did not provide detailed information about the proportion of the two components. This dog was consequently excluded from the calculation of fat, protein and, carbohydrate contents of the diet.

In order to aid comparison between the different types of dog food, the intake of protein, fat, NFE, fiber, and ash was calculated with a scientific computer program containing a nutrient database (for example different sorts of meat and offal), as used previously [3]. This computer program does also provide the possibility to add new feed types based on manufacture`s information regarding the nutrient content of their products. Practical experiences show that Weender analysis of BARF products is sometimes wrongly declared, resulting in a value of more or less than 100%, when summing the different contents (crude protein, fat, ash, fiber, NFE, and moisture). In these cases, nutrient data was directly substituted from the database, using meat sources with a similar fat content. Furthermore, some owners or diet manufactures could only give approximate information about the fat and protein content of their meat due to variation of each single batch. In these cases, an average was calculated. In addition, there was not always detailed data about the nutritional content available for all kinds of offal or bones used in the diets, so data of similar animals or similar types of bones were used. Treats were not taken into consideration because of varying amounts per day and incomplete data for precise quantities.

### Fecal collections

Owners were instructed to collect naturally passed fecal samples. The fecal samples were either brought to the clinic by the owner as soon as possible or picked up with a cool box at the owners’ homes and taken to the small animal clinic within a maximum of 12 hours. Owners living far away from the clinic were allowed to collect fecal samples the day before, place them in a fecal tube, freeze them over night and store the samples in a cool box with cooling elements during transport. Generally, owners were instructed to collect samples without contamination. Owners placed the samples in a dog waste bag, closed it airtight and clinic staff members transferred samples into fecal tubes. The stool sample of each dog was stored at—80°C until processing. All samples were homogenized before further analyses.

### DNA extraction and 16S rRNA gene sequencing

The DNA was extracted from 100 mg of each stool sample using a Mo Bio PowerSoil® DNA isolation kit (Mo Bio Laboratories) following the manufacturer`s instructions. The V4 region of the 16S rRNA gene was sequenced at MR DNA (www.mrdnalab.com, Shallowater, TX, USA). Briefly, using Primers 515F/806R and HotStarTaq Plus Master Mix (Qiagen, USA), samples were amplified in a 28-cycle PCR with the following protocol as used before [13]: 3 minutes 94°C, 30 seconds at 94°C (28 cycles), 40 seconds at 53°C, 1 minute at 72°C followed by 5 minutes at 72°C. Using Illumina TruSeq DNA`s protocol, a DNA library was set up and for further examination Illumina MiSeq was used for sequencing. Afterwards raw sequences were uploaded to the NCBI GenBank database under the accession number SRP117358.

### Analysis of sequences

As described before [13], QIIME v1.9 (Quantitative Insights Into Microbial Ecology) was used for further analysis and sequence data was demultiplexed. Using default settings for QIIME, low quality results were detected and removed. Furthermore, USEARCH was used to identify and eliminate chimeric sequences. The remaining sequences were assigned to operational taxonomic units (OTUs) by using an open reference approach in QIIME against the 97% clustered sequences from the Greengenes database.

Alpha diversity was evaluated with Chao 1, Shannon diversity, and observed species. Beta-diversity was evaluated by weighted an unweighted UniFrac distance matrices and visualized using PCoA (Principal Coordinate Analysis) plots. To describe which bacterial taxa and genes were associated with BARF or commercial diets, LEfSe (Linear discriminant analysis effect size) was used.

### Analysis of metabolites

To investigate differences in the fecal metabolome between BARF and commercially fed dogs, fecal samples from 10 BARF and 9 commercially fed dogs were used for further analysis. The sample number was reduced because of the cost associated with untargeted metabolomics. Samples were selected randomly from each group and also based on availability of sufficient fecal material. Samples were analyzed via gas chromatography time-of-flight mass spectrometry (GC-TOF-MS) as previously described [13, 14]. After obtaining 10 mg of a lyophilized sample, homogenization and extraction followed. After centrifugation, the supernatant was resuspended in methanol/chloroform. After adding internal standards derivatization by methoxyamine hydrochloride and N-methyl-N-trimethylsilyltrifluoroacetamide was used.

For detection, a temperature-gradient programmed gas chromatograph (oven 50°C to 330°C at 20°C/min, injector 50°C to 250°C at 12°C/sec) coupled with a helium carrier gas containing Leco Pegasus IV mass spectrometer (scanning 70 spectra/sec from 80–500 Da, -70 eV ionization energy, 1800 V detector voltage) was used. For detection 0.5 μl of the sample was injected onto a Restek rtx5Sil-mass spectrometry column using splitness mode. To process raw data files, ChromaTOF v. 2.32 software was used and results were uploaded to metabolomicsworkbench.org. For further assessment, the data table was filtered for metabolites of unknown identity which were excluded and afterward peak heights were uploaded to MetaboAnalyst 3.0, followed by log transformation and Pareto scaling.

### Quantitative real-time PCR (qPCR)

A qPCR was performed for selected bacterial groups: *Faecalibacterium* spp., *Fusobacterium* spp., *Blautia* spp., *Streptococcus* spp., *E*. *coli*, *Clostridium hiranonis*, *Turicibacter*, and *Campylobacter* spp. A CFX 96 Touch ^TM^ Real-Time PCR Detection system (Biorad Laboratories) was used for examination of the samples. As described previously [14–16], 10 μL TaqMan^®^ reaction mixtures (5 μL of TaqMan^®^ Fast Universal PCR master mix (2×), No AmpErase^®^ UNG (Applied Biosystems), 1 μL of water, 0.4 μL of each primer (final concentration: 400 nM), 0.2 μL of the probe (final concentration: 200 nM), 1 μL of 1% bovine serum albumin (BSA, final concentration: 0.1%), and 2 μL of DNA (1 : 10 or 1 : 100 dilution)) was used for further examination, following a protocol of 95°C for 20 s, and 40 cycles at 95°C for 5 s, and 10 s at the optimized annealing temperature. Ten μL SYBR-based reaction mixtures (5 μL of SsoFast^™^ EvaGreen^®^ supermix (Biorad Laboratories), 1.6 μL of water, 0.4 μL of each primer (final concentration: 400 nM), 1 μL of 1% BSA (final concentration: 0.1%), and 2 μL of DNA (1 : 10 or 1 : 100 dilution)) were used for a protocol of 95°C for 2 min, and 40 cycles at 95°C 5 s and 10 s at the optimized annealing temperature. Afterwards a melt curve analysis was set up. *Clostridium perfringens* was analyzed using a probe based protocol as described previously [17].

The qPCR results for *Faecalibacterium* spp., *Fusobacterium* spp., *Blautia* spp., *Streptococcus* spp., *E*. *coli*, *Clostridium hiranonis*, and *Turicibacter* were used to calculate the degree of dysbiosis in feces of BARF and commercially fed dogs. The changes in these bacterial groups are summarized in the Dysbiosis Index (DI). A negative value shows a normal microbiota, a positive value indicates dysbiosis [16].

### Statistical analysis

To analyze dog characteristics and nutrient composition of the diets, a t-test was performed. If normality (alpha = 0.05) was not passed, a Mann-Whitney-test was used for statistical analysis. The web-based program Calypso was used to visualize the generated OTU tables, while ANOSIM was used to show differences between microbial communities between the two groups. Mann-Whitney-test was used to compare bacterial taxa and metabolic compounds between the BARF and the commercial fed dogs. P-values were adjusted for multiple comparisons using the Benjamini and Hochberg False discovery rate and significance was set at q < 0.05. Linear discriminant analysis effect size was used to visualize bacterial taxa different between BARF and commercially fed dogs. The LDA score was set at ≥ 3.0. Furthermore, random forest analysis was used to detect the impact of different metabolites between both groups.

## Results

### Animal population

Table 1 summarizes the characteristics of the BARF (n = 27) and control group (n = 19). There was no significant difference in gender, age, and body weight. Breeds used in the study and body condition scores are summarized as supplementary data (S1 Table).

**Table 1:** Dog characteristics.

	BARF	commercial	p—value
Gender (male/female)	12/15	9/10	> 0.9999
Age in years (median/range)	4/0.58–15	5/2–15	0.3410
Weight in kg (median/range)	21/2–50	13.2/6.5–30	0.9339

### Diets

**Fig 1:**
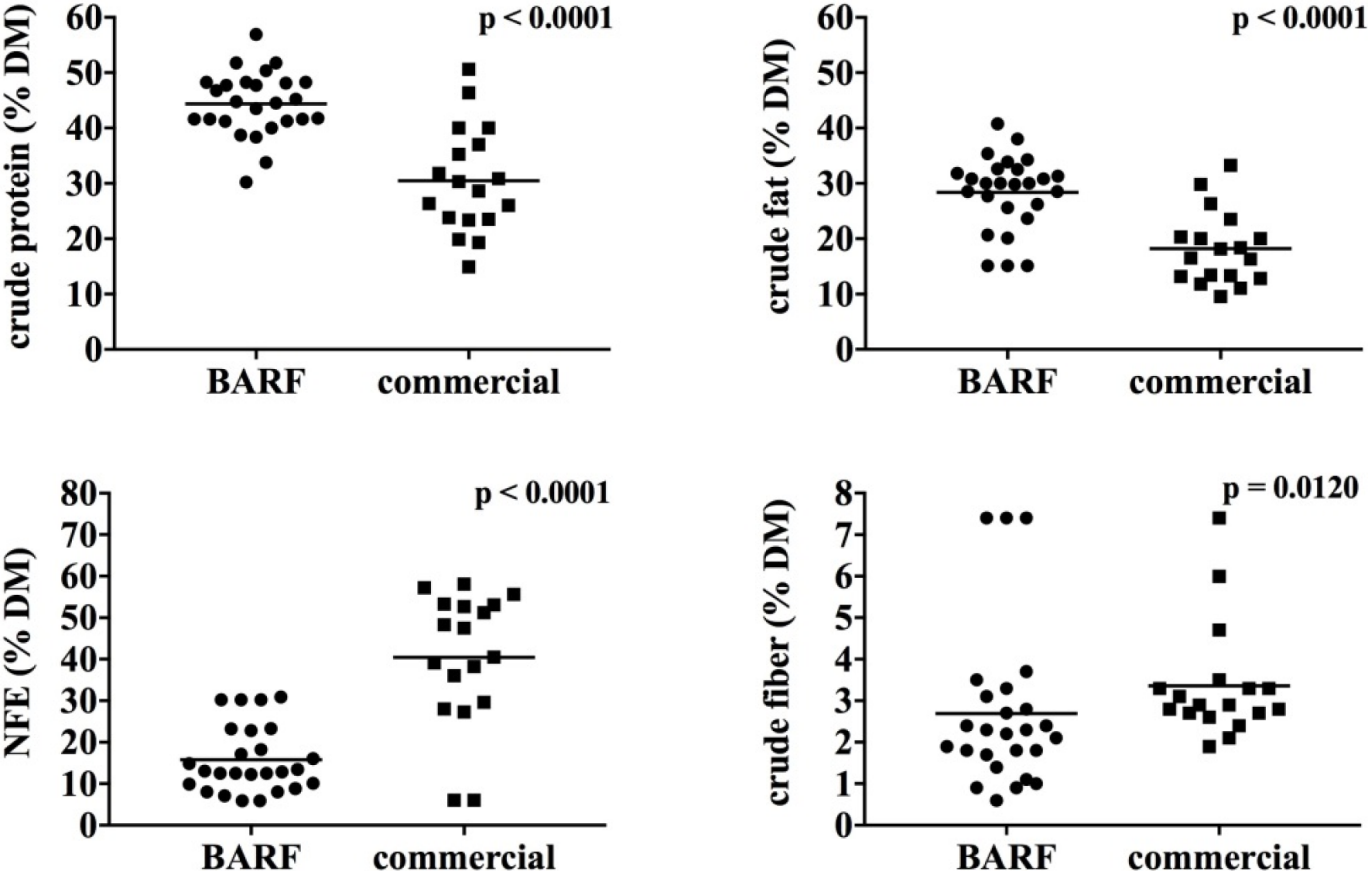
Intake of crude protein, fat, fiber, and NFE (Nitrogen-Free Extract) between both groups. The BARF dogs were fed a significantly higher amount of protein and fat and lower amount of NFE and fiber.

Evaluation of diets showed a significant difference in protein, fat, carbohydrate, and fiber intake between the two groups (Fig 1). The BARF dogs were fed a higher amount of protein (mean 44.40 ± 5.80 Standard Deviation (SD) in % DM) caused by a high content of animal products like meat, offal, and bones in the diet. Furthermore, fat in the form of marbled meat, animal fat and fish- or vegetable oil played an important role as energy source (28.40 ± 6.72 in % DM) in BARF diets, whereas carbohydrates were commonly only used in small amounts or infrequently as energy source (15.75 ± 7.99 in % DM). The fiber intake was significantly lower in BARF dogs (2.69 ± 1.92 in % DM). Three dogs of the BARF group were fed hay cobs which resulted in a higher intake of crude fiber compared to the other dogs.

In contrast, dry fed dogs or dogs who were fed a combination of dry and wet food, ingested more carbohydrates (mean 40.43 ± 16.03 SD in % DM), which are commonly used as energy source in dry dog food, resulting in a comparatively lower protein and fat intake. The mean protein content of the diet was 30.45 ± 9.61 in % DM, the fat content was 18.21 ± 6.60 SD in % DM and the fiber intake 3.36 ± 1.38 in % DM.

### Effect of BARF vs. commercial food on gut bacterial diversity

#### Diversity of microbial community

**Fig 2:**
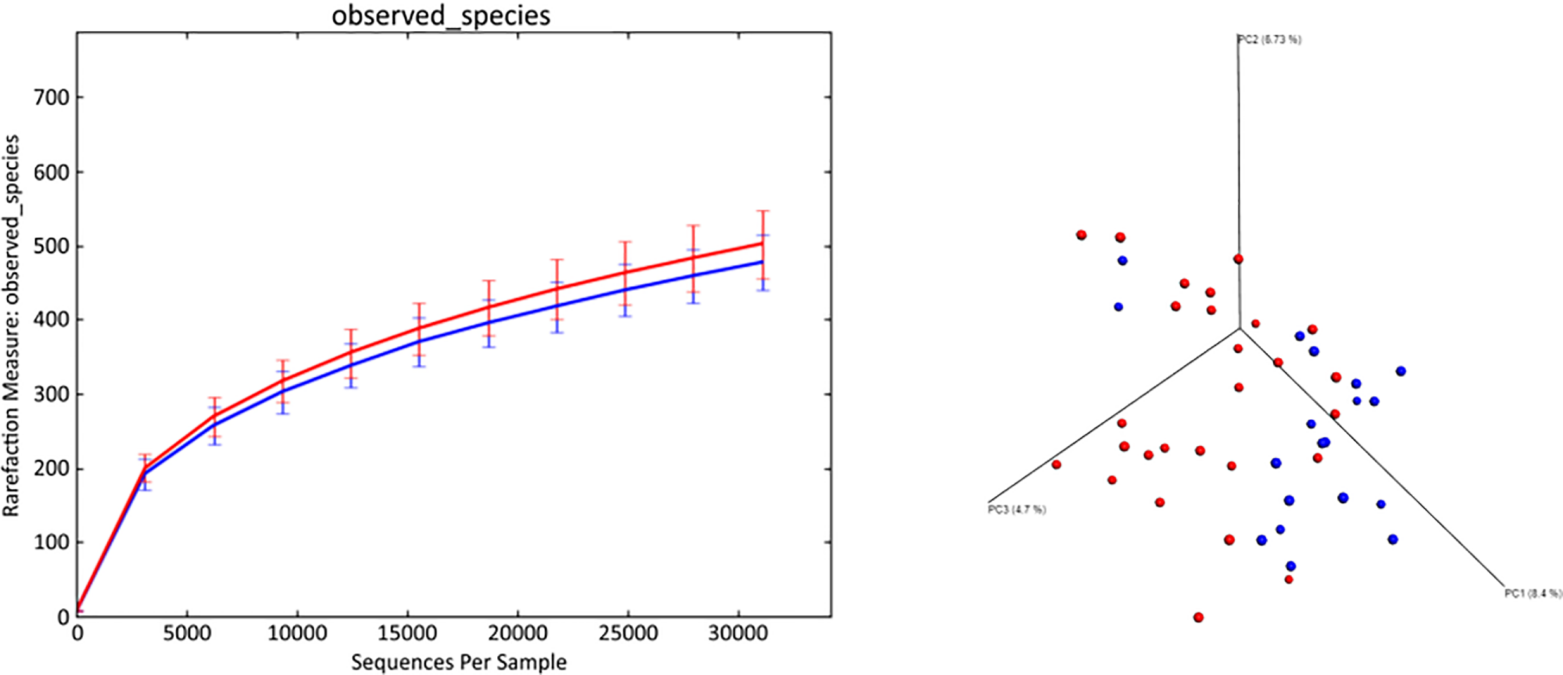
Bacterial diversity measures. (a) Alpha diversity: Rarefaction analysis of 16S rRNA gene sequences in raw and commercially fed dogs. Lines represent the mean of each group (red = BARF, blue = commercial), the standard deviation is shown by error bars. No significant difference in observed species was seen between BARF and commercially fed dogs. (b) Beta diversity: Principal coordinates analysis (PCoA) plot showing clustering of microbial communities from feces of raw and commercially fed dogs (red = BARF, blue = commercial). The closer the items, the more similar is the microbial community of the samples. Consequently, the microbiome of BARF dogs differs from the microbiome of commercially fed dogs (ANOSIM; p < 0.01).

Table 2 shows the results of alpha diversity measures. No significant differences in Chao 1, Shannon diversity index, and species richness were seen. Fig 2A shows the rarefaction analysis of 16S rRNA gene sequences for both groups.

**Table 2:** Summary of alpha diversity measures.

	BARF		commercial			
Diversity	mean	standard deviation	mean	standard deviation	p-value	q-value
Chao1	1595	171	1559	192	0.655	0.655
Observed_species	800	68	759	78	0.086	0.296
Shannon Index	5.07	0.44	4.82	0.61	0.148	0.296

Principal coordinate analysis (PCoA) plots were used to compare microbial communities of BARF and commercially fed dogs. The BARF dogs showed a significantly different microbiome compared to commercially fed dogs, as shown in Fig 2B (ANOSIM; p < 0.01).

#### Bacterial taxa of BARF vs. commercially fed dogs

Several differences in abundance of bacterial taxa between the two diets were detected. Linear discriminant analysis (LDA) effect size (LEfSe) was used and results compared with univariate statistics (S2 Table).

Linear discriminant analysis effect size identified 34 bacterial taxa which were significantly different between the two groups (Table 3). Using a LDA score ≥ 3 as cut-off, BARF dogs showed a higher abundance of *Lactobacillales* on an order level, as well as *Enterobacteriaceae* on a family level, and *Fusobacterium*, *Clostridium* and *Enterococcus* on a genus level. Conversely, commercial fed dogs had a higher abundance of *Clostridiaceae*.

**Table 3:** Linear discriminant analysis of bacterial taxa and their associations with diet. Only an LDA score of ≥ 3.0 is shown.

	Diet	LDA
g__*Fusobacterium*	BARF	4.49
g__*Clostridium*	BARF	4.34
f__*Enterobacteriaceae*.g__	BARF	4.31
o__*Lactobacillales*.f__.g__	BARF	4.01
g__*Enterococcus*	BARF	3.74
g__*Trichococcus*	BARF	3.62
f__*Moraxellaceae*	BARF	3.52
o__*Gaiellales*.f__AK1AB1__02E.g__	BARF	3.34
o__*Gaiellales*	BARF	3.33
f__*Hyphomicrobiaceae*.g__	BARF	3.32
f__*Micrococcaceae*.g__	BARF	3.31
f__*Lactobacillaceae*.g__	BARF	3.29
f__*Sinobacteraceae*.g__	BARF	3.23
g__*Uliginosibacterium*	BARF	3.21
o__*Rhodocyclales*	BARF	3.13
g__*Gemella*	BARF	3.09
g__*Leuconostoc*	BARF	3.09
o__*Lactobacillales*.Other.Other	BARF	3.00
f__*Clostridiaceae*.Other	commercial	4.69
f__*Clostridiaceae*.g__	commercial	3.89
g__*Catenibacterium*	commercial	3.83
f__*Lachnospiraceae*.Other	commercial	3.78
g__*Faecalibacterium*	commercial	3.75
g__*Eubacterium*__	commercial	3.65
g__*Devosia*	commercial	3.46
g__*Steroidobacter*	commercial	3.39
f__*Erysipelotrichaceae*.g__	commercial	3.34
g__*Lachnospira*	commercial	3.33
g__*Bifidobacterium*	commercial	3.18
f__*Chthoniobacteraceae*__	commercial	3.13
c__*Spartobacteria*__	commercial	3.09
o__*Opitutales*	commercial	3.09
o__*Chthoniobacterales*__	commercial	3.04
c__*Opitutae*	commercial	3.01

Taxonomic levels are represented as c (class), o (order), f (family), and g (genus)

On univariate statistics, feeding a BARF diet had significant effect on the presence of *Proteobacteria* and *Fusobacteria*, which were higher in the BARF group (p < 0.0001 and p = 0.013), whereas *Firmicutes* had a higher abundance in commercially fed dogs (p = 0.001).

At the family level *Enterobacteriaceae* (p < 0.0001), *Methanobacteriaceae* (p = 0.004), *Lactobacillales* (p = 0.002) and *Carnobacteriaceae* (p = 0.004) were increased, while *Bifidobacteriaceae* (p = 0.003), *Ruminococcaceae* (p = 0.007) and *Erysipelotrichaceae* (p = 0.003) were significantly lower in the group of BARF dogs, compared to commercial fed dogs.

At the genus level *Methanobrevibacter* (p = 0.007), *Carnobacterium* (p = 0.005), *Clostridium* (p = 0.003), and *Cellvibrio* (p < 0.0001) were higher represented in the feces of BARF dogs, whereas *Bifidobacterium* (p = 0.004), *Epulopiscium* (p = 0.006), *Erysipelotrichaceae* (p = 0.002) and *Faecalibacterium* (p = 0.002) were higher in the control group.

Predominated bacteria at different taxonomic levels are shown in S1 Table. Consequently, the results of univariate statistics were quite similar to the outcome of LEfSe.

#### qPCR and Dysbiosis Index

**Fig 3:**
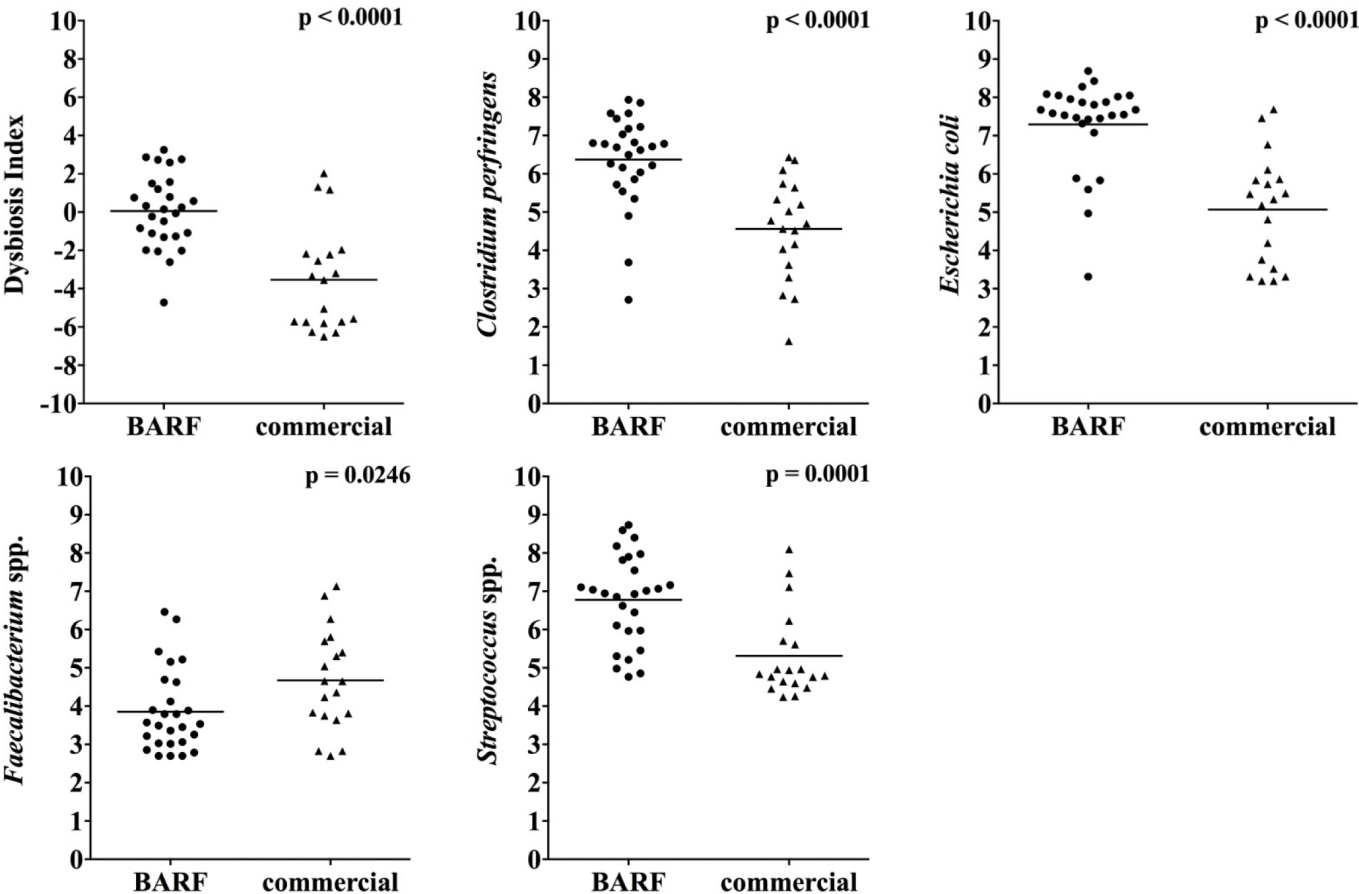
Fecal abundance of selected bacterial taxa between BARF and commercially fed dogs based on qPCR. **The** BARF dogs showed a higher abundance of *Clostridium perfringens*, *E*. *coli* and *Streptococcus*, while commercially fed dogs showed a higher abundance of *Faecalibacterium*. The Dysbiosis Index was significantly higher in the BARF group.

Analysis by qPCR showed a higher abundance of *Clostridium perfringens* (p < 0.0001), *Streptococcus* (p = 0.0001) and *E*. *coli* (P < 0.0001) in the BARF group, whereas *Faecalibacterium* was lower (p = 0.026) compared to the commercially fed dogs. No significant differences between the two groups were seen in the abundance of *Fusobacterium*, *Campylobacter*, and *Blautia*. The Dysbiosis Index was significantly higher in the raw fed dog group (p < 0.001), which was driven by a higher abundance of *E*. *coli* (p < 0.0001) and lower abundance in *Faecalibacterium* (p < 0.025) (Fig 3).

### Effect of BARF on fecal metabolomics

Altogether 233 different metabolites were identified. To detect and visualize differences in the fecal metabolome, principal component analysis (PCA), random forest analysis and univariate statistics were used, followed by an adjustment for multiple comparison.

**Fig 4:**
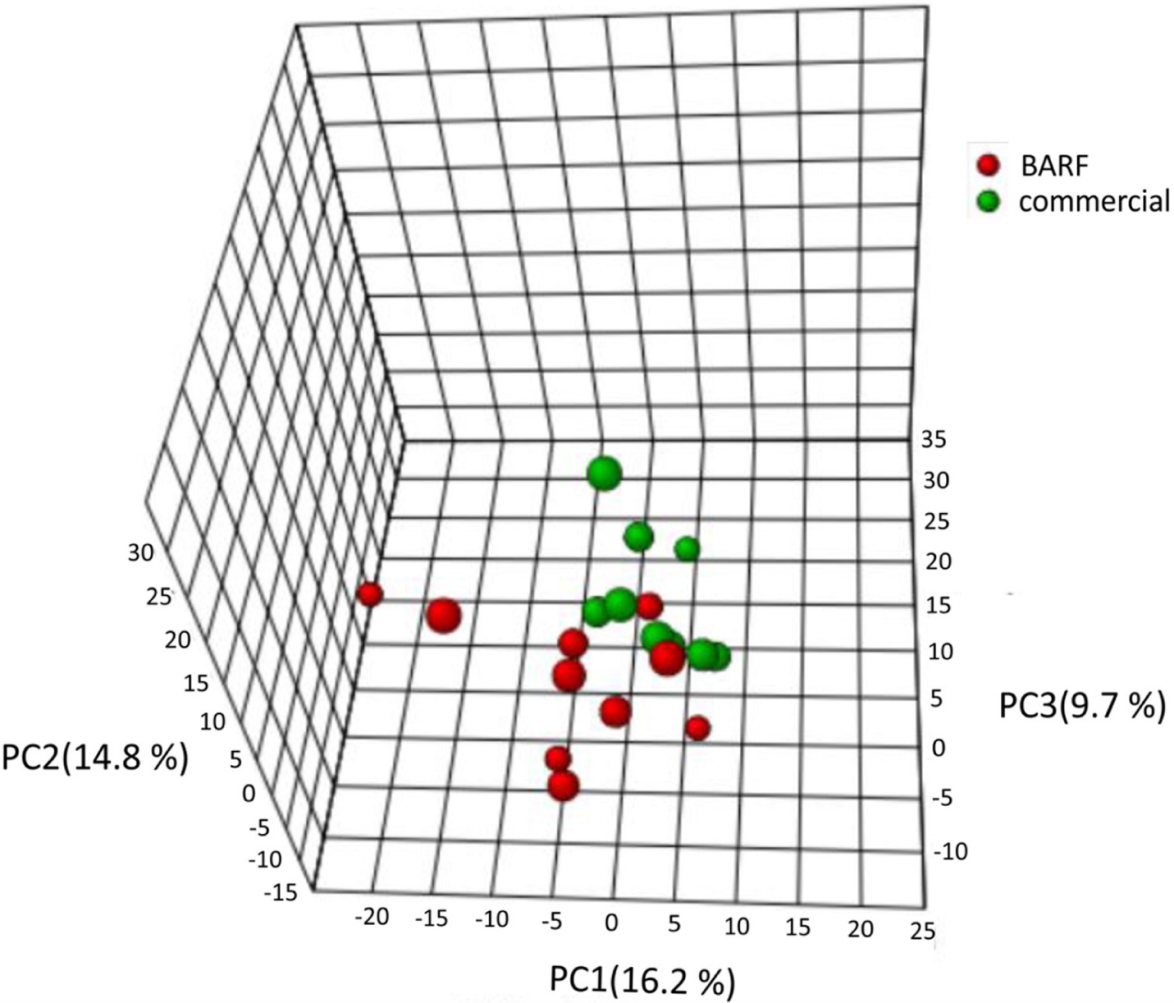
PCA plot of the fecal metabolome showing clustering of samples based on BARF versus commercially fed diets. The BARF (red) and commercially (green) fecal samples were used to detect differences between the two diets. The PCA plot showed a clustering between both groups.

As shown in Fig 4, PCA plots showed a clustering of samples based on BARF versus commercially fed diets.

**Fig 5:**
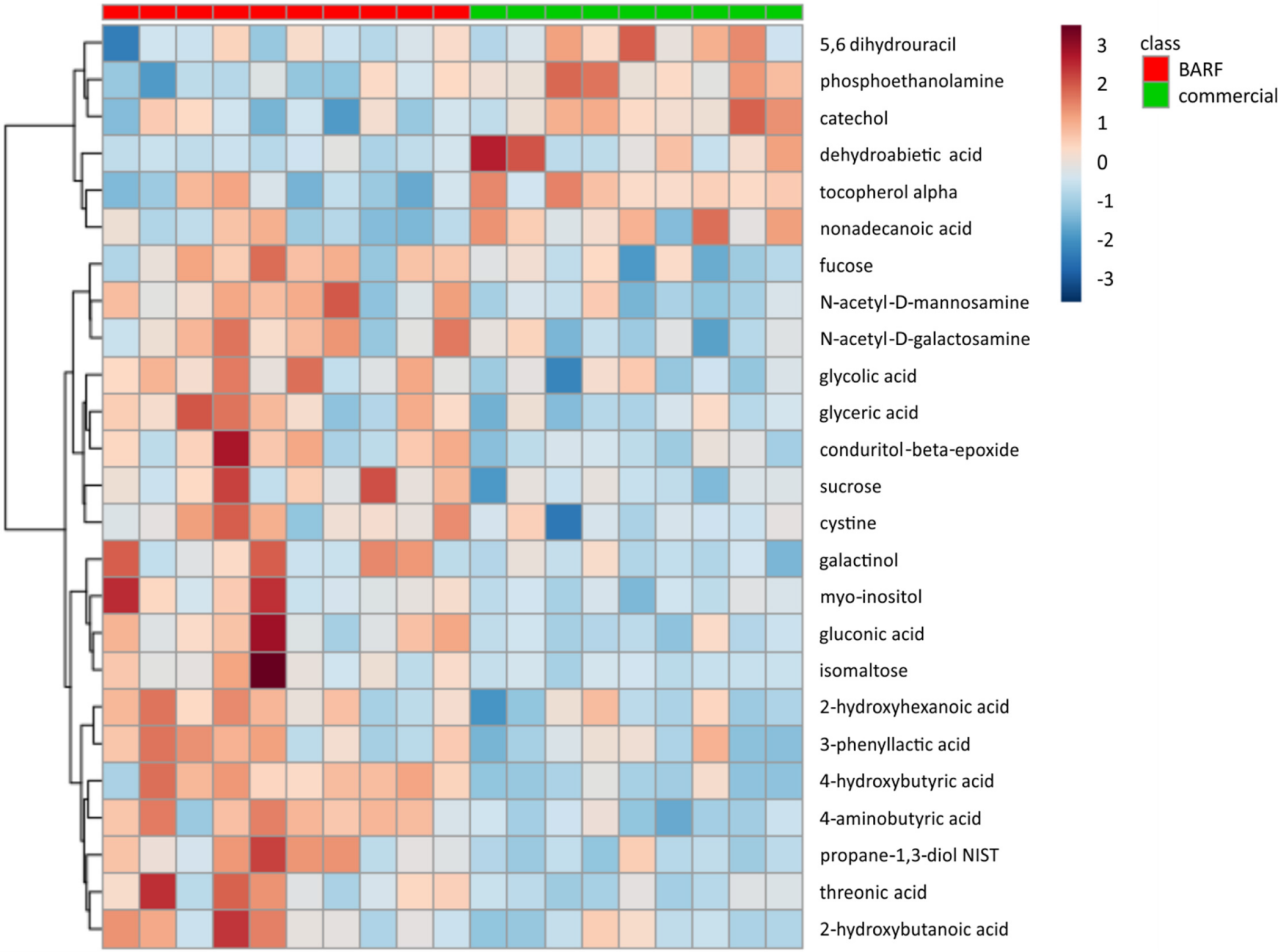
Heatmap of the most abundant metabolites of both groups, as identified by VIP scores in PLS-DA. Every sample (10 BARF samples = red, 9 commercial samples = green) is represented by an own column, the more red colored the higher the abundance of these metabolites. The BARF dogs showed a higher abundance of e.g. myo-inositol, gluconic acid or isomaltose, the commercially fed dogs a higher abundance e.g. of catechol or phosphoethanolamine.

To summarize the distribution of the most significant metabolites separating the two diet groups, a heat map was used to visualize the results of the major abundant metabolites (Fig 5). Every column represents a different metabolite in one stool sample. Higher abundances are marked in red color, whereas lower abundances in blue color. The BARF group showed a higher abundance of myo-inositol, gluconic acid, isomaltose, 4-hydroxybutyric acid, 4-aminobutyric acid and threonic acid, the commercially fed group a higher abundance of 5,6 dihydrouracil, phosphoethanolamine, catechol, alpha-tocopherol and dehydroabietic acid.

**Fig 6:**
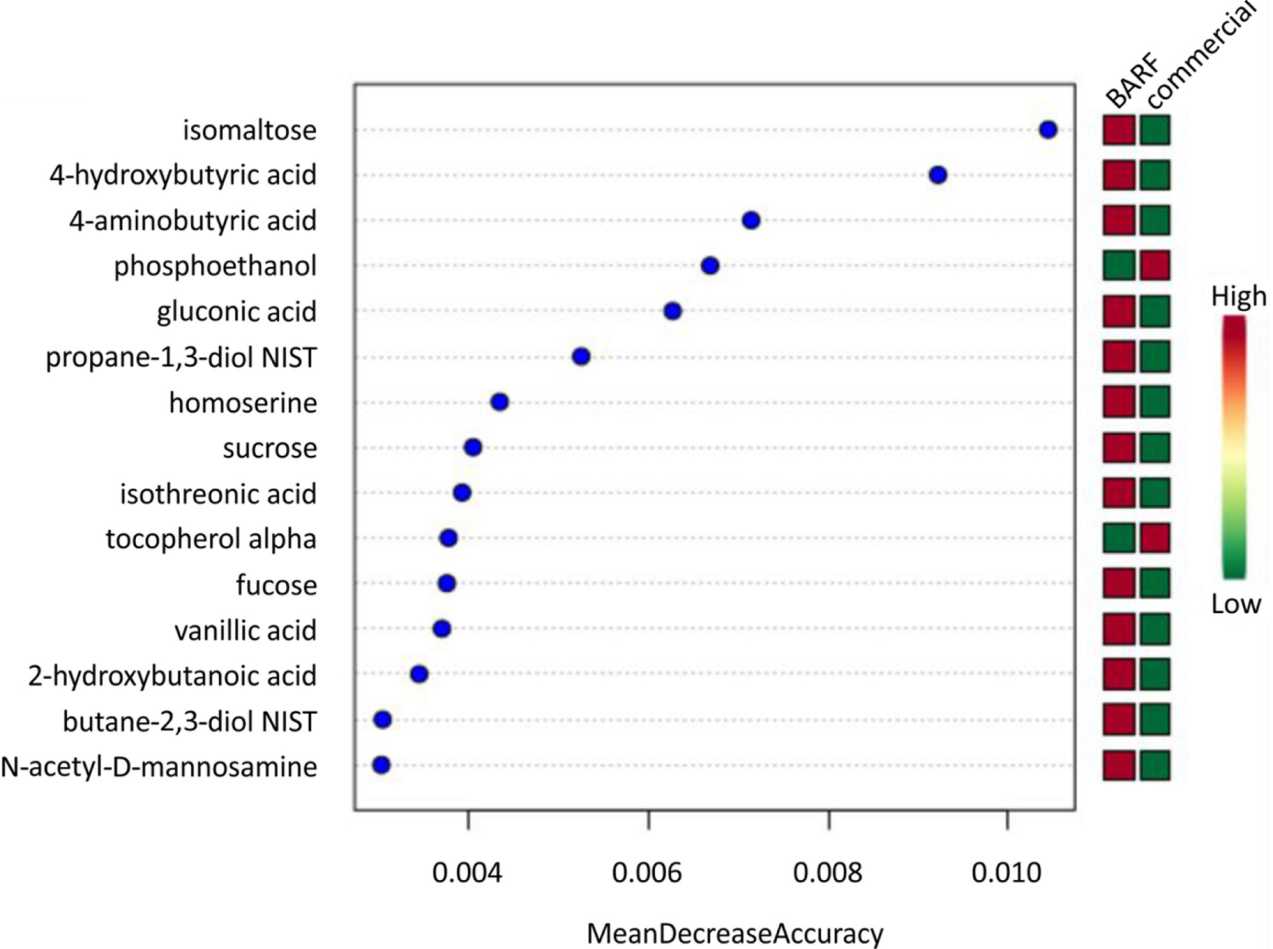
Random forest analysis. Top 15 metabolites with the highest discriminatory power between both diet groups are listed. Red fields show a high abundance, green fields a low abundance of the particular metabolite based on diet.

Furthermore, Random Forest Analysis was used to find metabolites with the highest discriminatory power between the two groups. In the BARF group isomaltose, 4-hydroxybutyric-acid, 4-aminobutyric-acid, gluconic acid, propane-1,3-diol NIST, homoserine, sucrose, isothreonic acid, fucose, vanillic acid, 2-hydroxybutanoic acid, butane-2,3-diol NIST, N-acetyl-D-mannosamine were the most important metabolites, in the commercially fed group phosphoethanol and tocopherol alpha (Fig 6).

Based on univariate statistics, thirty-three of 233 metabolites were significantly different on unadjusted p-values between the diets (p < 0.05), e.g., a higher abundance of 4-hydroxybutyric-acid, isomaltose and myo-inositol in the BARF group and a higher abundance of and tocopherol alpha and phosphoethanolamine in the commercially fed group. All these metabolites lost significance after adjustment for multiple comparisons (q > 0.05).

**Fig 7:**
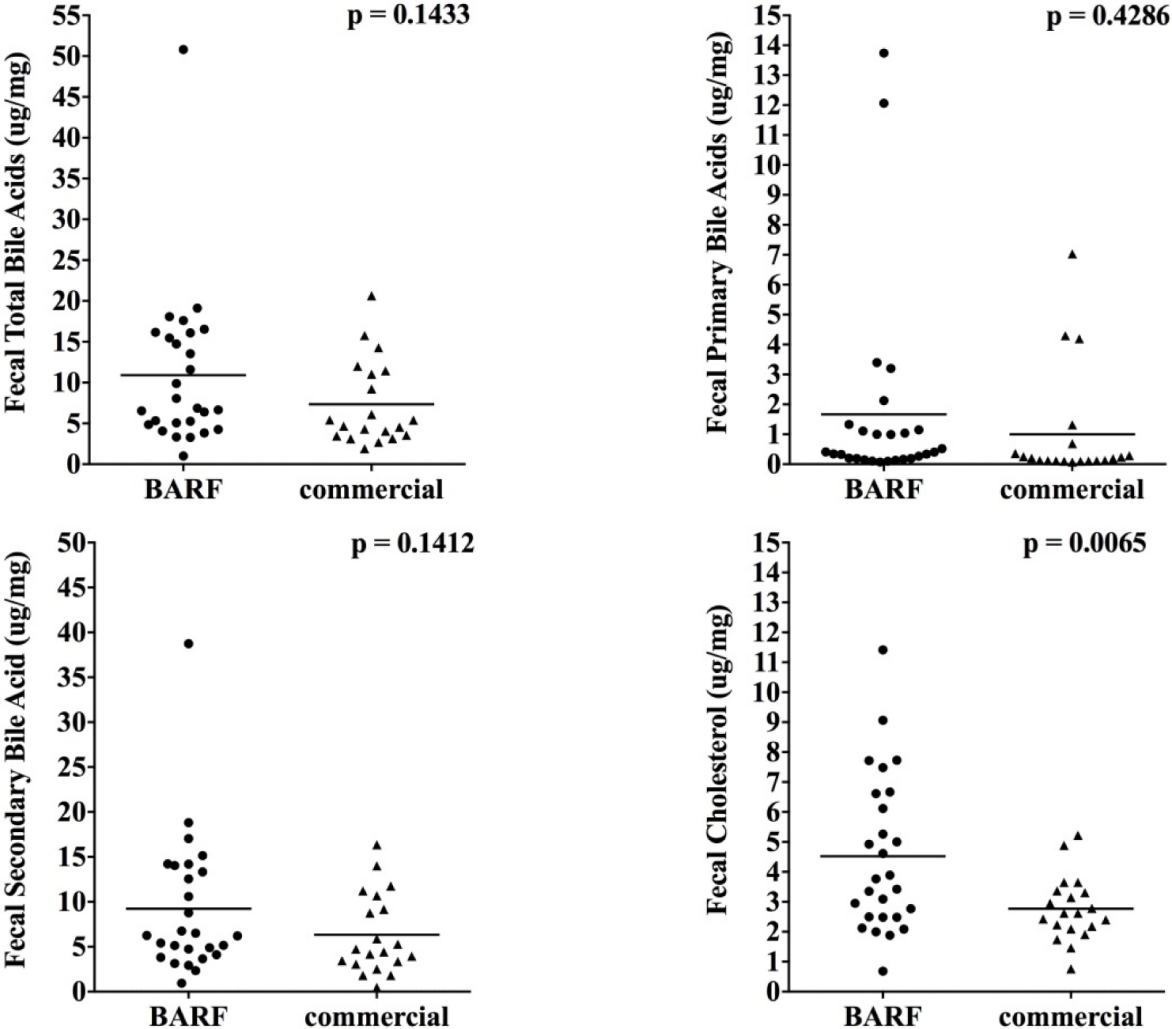
Total, primary, secondary fecal bile acids and cholesterol of BARF and commercially fed dogs. There was no significant difference in total, primary, and secondary bile acids between fecal samples of both groups, but BARF dogs had a higher abundance of cholesterol in their feces.

Fig 7 shows that fecal cholesterol was increased in the fecal samples of BARF fed dogs (p = 0.0065), whereas primary, secondary and total fecal bile acid concentrations did not differ significantly.

## Discussion

Several studies revealed the potential risk of transmission and excretion of pathogens via contaminated meat [18, 19], but only a few studies have investigated effects of feeding raw meat on the microbiome of dogs [11, 12, 20]. The current trial indicates that a profound dietary change, especially with major changes in protein and fat content, can influence the gut microbiome considerably. This result is also confirmed by other findings, for example in a feeding trial by Bermingham et al. [21]. In this survey, cats were exposed to a dry diet (crude protein 33.0%, fat 11.0%, carbohydrate 49.4%, ash 6.6% in % DM) for five weeks after feeding a wet diet with significantly altered macronutrients (crude protein 51.7%, fat 28.9%, carbohydrate 8.9%, ash 10.6% in % DM) for a period of five weeks. This switch in the diet led to numerous alterations in the abundance of bacterial taxa like *Fusobacteriaceae* and *Pelomonas* [21]. In 2013, Bermingham et al. [22] compared two groups of cats again: one group was fed a dry diet (32.9% crude protein, 11.1% fat, 45.9% carbohydrates, 8.3% ash, crude fiber 1.9% in % dry matter (DM)), the other one with a wet diet (41.9% protein, 42.4% fat, 5.3% carbohydrates, ash 8.8%, crude fiber 1.6% in % DM). The results revealed significant effects on the fecal bacterial population, e.g., a higher amount of *Actinobacteria* and a lower abundance of *Proteobacteria* and *Fusobacteria* in the dry food group [22].

Minor modifications of the diet, however, do not lead to consistent changes in the microbiota, as shown for example by Panasevich et al. 2017 [7]. An addition of up to 6% potato fiber increased the amount of *Firmicutes* and decreased *Fusobacteria*, but the changes were rather small in total. Similar effects were also seen in a recent study comparing effects of diet and antibiotics: while metronidazole had a profound effect on the fecal microbiome and metabolome, a switch from a canned or dry food diet to a hydrolyzed protein diet of similar macronutrients did not affect the microbiome significantly [9].

To investigate the effect of raw meat based diets vs. extruded diets on the fecal microbiome, Sandri et al. [11] performed a feeding trial with two groups of dogs being either fed a raw based diet (26.2% crude protein, 18.2% crude fat, 50.6% NFE, 0.7% crude fiber, 4.3% ash in % DM) or a commercial diet with a similar formula (26.7% crude protein, 10.6% crude fat, 49.9% NFE, 2.8% crude fiber and 10.0% ash in % DM), respectively. This modification in the diet led to a significantly higher abundance of *Proteobacteria* and *Fusobacteria* in dogs fed the raw based diet, which was also found in our current study. However, dogs being fed a raw diet in Sandri`s trial did not display a significant change in the abundance of *Firmicutes*, while dogs in the current trial showed significantly lower levels than conventionally fed dogs. Regarding the abundance of *Actinobacteria*, dogs on a raw based diet in Sandri`s study showed higher levels, while there was no significant difference between both groups in our trial. Both studies differ regarding the composition of the BARF diets as dogs in Sandri`s study were fed beef meat and highly digestible carbohydrates, while most dogs in the current study received a combination of muscle meat, offal, and partially raw meaty bones. Furthermore, protein contents were higher and carbohydrate contents lower (44.4% protein, 15.8% NFE) in the current study compared to Sandri`s trial.

On a family level, a significantly higher abundance of *Enterobacteriaceae* in the current BARF group was also shown by Sandri et al. [11]. A significant decrease of *Ruminococcaceae* in raw fed dogs as shown in this study was also reported from Bermingham et al. [12]. Moreover, the finding of a higher abundance of *Erysipelotrichaceae* in the control group of the current study has also been shown previously [12, 23].

On a genus level, dogs on a BARF diet revealed a significantly higher abundance of *Clostridium* in the current study. Furthermore, qPCR did also detect a significant difference in the abundance of *Clostridium perfringens* in dogs fed a BARF diet. The effect of feeding high protein diets on an increase of *Clostridium* or *Clostridium perfringens* has been documented previously in several studies [12, 22–24].

Furthermore, qPCR results showed a higher abundance of *E*. *coli* in the BARF group. While *E*. *coli* are normal commensals in the gut and most strains are nonpathogenic, *E*. *coli* has the ability to attach to the intestinal wall and may lead to gastroenteritis in some animals [25, 26]. The health risk for dogs ingesting *E*. *coli* has not been fully clarified yet, and a potential contamination of BARF-food has been confirmed in different studies [27, 28]. Furthermore, several feeding trials have examined the influence of different dietary protein contents on the abundance of *E*. *coli*. Lubbs et al. found out that feeding a high-protein (52.9% crude protein in % DM) vs. a moderate-protein (34.3% crude protein in % DM) diet did not affect the abundance of *E*. *coli* in adult cats [29] or did even lead to a lower abundance of *E*.*coli* in growing kitten on the same diet as shown in a study of Vester et al. [30]. However, feeding a high protein diet increased the abundance of *E*. *coli* in the current study. A similar effect was also revealed in a trial with two groups of rats, one fed with a diet containing 20.1% protein and 55.5% carbohydrates in % DM, and the other group fed with a diet of 45.1% protein and 29.9% carbohydrates in % DM [31]. Furthermore, a significant reduction of *E*. *coli* in the feces of dogs was demonstrated in a study of Gonzàles-Ortiz et.al, in which dietary protein was reduced from 16.7 g to 8.37 g crude protein/MJ [32]. To rule out a mutual effect between absorption of *E*. *coli* via food and the type of diet, a comparison of the abundance of fecal *E*. *coli* in BARF and canned fed dogs might be useful, as both types of diet provide high amounts of dietary protein.

Interestingly, there was no considerable difference in the abundance of *Fusobacterium* between BARF and commercially fed dogs in qPCR, even though LefSe showed a significant higher abundance of *Fusobacterium* in the BARF diet (LDA 4.49). An explanation for this discrepancy is yet to be found. This divergence emphasizes once more the usefulness of a combination of different detection methods and a critical evaluation of data collected.

The Dysbiosis Index DI was significantly different between both groups. This index was trained against the microbiota of dogs with chronic intestinal inflammation [16]. The major bacterial taxa that are contributing to intestinal dysbiosis in dogs are increases in *E*. *coli* and *Streptococcus*, and decreases in *Faecalibacterium* [14, 33–35]. In this study we showed that feeding a high protein and high fat diet significantly decreased *Faecalibacterium* and increased *Streptococcus*, *E*. *coli*, and *C*. *perfringens*, the last group also often seen increased in GI disease [35]. Our understanding of the contributions of the microbiota to chronic intestinal diseases is still developing. It is likely that several factors (i.e., genetic background of the host, damage to intestinal epithelium due to environmental triggers) together with alterations in the intestinal microbiota need to interact to induce disease. Therefore, at this stage it is unknown, whether the changes observed in this study will cause intestinal diseases in the future. Nevertheless, our study was able to show that typical BARF diets may induce some of these alterations in the intestinal microbiota.

The influence of diet on the fecal metabolome is rarely investigated. As summarized in the results section, PCoA plots showed a clustering of the fecal metabolome of BARF versus commercially fed dogs, and random forest analysis showed various differences between both groups. Furthermore, univariant analysis identified several compounds that were significantly different based on unadjusted p-values, but not after adjusting p-values. At this point the best cut-off value is not clearly determined. These results together indicate that the fecal metabolome differs between both groups.

Studies have previously investigated the effect of diet and disease on the abundance of cholesterol, primary and secondary bile acids. These metabolic compounds demonstrate that host and intestinal microbiota operate very closely, as colonic bacteria are necessary for the conversion of primary to secondary bile acids. In intestinal dysbiosis due to gastrointestinal disease, alterations in fecal bile acid concentrations, especially a reduction of fecal secondary bile acids due to an inadequate bacterial conversion has been reported [36, 37]. While a significant alteration in the abundance of cholesterol could not be found in dogs with IBD [36], human patients with ulcerative colitis excreted a higher amount of cholesterol, whereas bile acid excretion was not significantly altered [38]. Less is known about dietary factors, and our results showed that BARF dogs did not show alterations in fecal bile acid concentrations, while fecal cholesterol was significantly increased. Different studies have shown that special fiber sources like sugar-beet fiber or oat bran as well as polyunsaturated fat can lead to a higher excretion of fecal cholesterol in human, rats and hamsters for example [39–41]. As the enrolled dogs in our study were in a good health state, blood values were not evaluated, and crude fiber content of the diets was quite low, further studies are needed to evaluate the difference in the abundance of cholesterol and bile acids in dogs fed different diet compositions.

Another fecal metabolite higher abundant in the BARF group was isomaltose. This disaccharide results from the degradation of dietary starch or glycogen by means of the enzyme alpha-amylase. Isomaltose is then further converted enzymatically into glucose. Being part of isomalto-oligosaccharides, isomaltose has prebiotic functions and can be used pharmaceutically due to its antimicrobial activity [42]. Nonetheless, isomaltose seems to play a role in several pathological processes. For example, deficiencies might result in starch intolerance and diarrhea [43, 44]. Furthermore, serious injuries or diseases can lead to an increased excretion of isomaltose via urine in humans [45, 46] or a higher abundance in plasma of patients with chronic renal diseases [47]. To the best of our knowledge, no study has examined the fecal excretion of isomaltose in dogs being fed different diets or dogs of different health states yet.

In our study we observed that BARF dogs showed a higher abundance of 4-hydroxybutyric acid (GHB) and 4-aminobutyric acid (GABA) in their feces. Gamma-aminobutyric acid is an important neurotransmitter with inhibitory character in the central nervous system. It seems that GABA can also be influenced by diet: as shown in a study of Olson et al. [48], feeding a ketogenic diet led to an alteration of the intestinal microbiota and an increase of hippocampal GABA/glutamate ratio. Gamma-hydroxybutyric acid being a short chain fatty acid and metabolite of GABA, might also act as neurotransmitter [49]. While the positive effect of special food components in human epilepsy-therapy is well known, especially in form of ketogenic diets for children [50], our knowledge about dietary intervention for dogs with epilepsy is still developing. Law et al. showed that feeding a ketogenic medium chain TAG diet (MCTD) lowered the seizure frequency of the enrolled dogs [51]. In another study Pan et al. discovered that supplementation of MCT led to a higher abundance of the ketone body beta-hydroxybutyrate as well as an improved cognition function in aged dogs [52]. Further studies will be needed to evaluate the effect of dietary intervention like adjusting fat, protein- and NFE contents and supplementation of fatty acids in dogs with epilepsy.

Dogs fed a BARF diet in our trial showed a higher abundance of gluconic acid than commercially fed dogs. This derivate of glucose is a nonvolatile mild organic acid that appears for example in meat, plants or dairy products [53]. Studies have shown that gluconic acid might have a prebiotic effect as it stimulates lactic acid bacteria and the production of butyrate in pigs [54] and *Bifidobacteria* in humans [55]. It needs to be clarified why BARF dogs show a higher abundance of this metabolite in our study.

It would have been useful to also include an analysis of fecal SCFA concentrations. However, SCFA are volatile compounds and the metabolomic platform used in this study does not allow for measurement of such volatile metabolites. The reason for choosing the current metabolomics platform is that it clearly expands our knowledge of additional metabolites that are crucial for host health and measurable in feces. Some of these metabolites (amino acids, lactate, bile acids) have been observed to be altered in feces of dogs with acute and chronic intestinal disease. This metabolomics approach may yield useful information about important biochemical pathways that may be altered with diet. Current limitation of metabolomics is the lack of a single platform that could allow a stringent measurement of the majority of these compounds.

There are some limitations in this study, including the ingestion of a widely varying intake of macronutrients. Especially canned and dry diets provide different amounts of protein, fat and NFE. In general, a commonality of both commercial diets is heating during the manufacturing process for long-term preservation and germ elimination, in contrast to meat in BARF diets, which are being fed raw and unprocessed. Because of this, both types of commercial food were included in the study. It is also important to note that fermentable and non-fermentable fiber sources were not differentiated in this trial. Another limitation is that dogs might also have eaten external material like feces of other animals or garbage in a moment being unobserved by their owners. Furthermore, dogs of different home environments and different breeds were included in this study and some samples were handled directly by the owners. Nonetheless, it is important to compare these various diets that are used by owners in their home environments.

## Conclusion

Different food composition altered the microbiota structure significantly, while microbiota richness was not significantly changed. The BARF dogs had a significantly higher DI driven by an increase in *E*. *coli* and *Streptococcus*, and a decrease in *Faecalibacterium*. Furthermore, *Clostridium perfringens* was significantly higher in BARF diets. Moreover, BARF diets had a strong influence on the metabolome: while primary and secondary bile acids were not significantly altered, fecal cholesterol was increased in the feces of BARF dogs. Several metabolites like isomaltose, GABA, and GHB were different between both groups as shown in PCoA plots, heat map and random forest analysis, nonetheless these components lost significance after adjustment using univariate statistics. The results suggest a notable influence of differences of the compositions of macronutrients on the fecal microbiome and metabolome. Further studies about metabolic effects of BARF diets are required for a better understanding of these effects on dogs.

## Supporting information

S1 Table Breeds and body condition scores of the enrolled dogs. (XLSX)

S2 Table Relative abundance of bacterial groups on different phylogenetic levels. (XLSX)

S1 File Infobox BARF. (DOCX)

S2 File Questions used in the study—German language. (DOCX)

S3 File Questions used in the study—English language. (DOCX)
