# Supplementary material for: The fecal microbiome and metabolome differs between dogs fed Bones and Raw Food (BARF) diets and dogs fed commercial diets
Source: PLoS One. 2018 Aug 15;13(8):e0201279. doi: 10.1371/journal.pone.0201279 (PMC6093636; doi:10.1371/journal.pone.0201279)
Supplement: S1 File — (DOCX) [file pone.0201279.s003.docx]

**S1 File. Infobox BARF**

To get a closer look at the composition of different BARF plans, components of ten BARF rations were considered more precisely. The distribution of animal- vs. plant-based ingredients was about 70 % vs. 30 %.

Two dog owners did not feed any bones, but in the mean, about one half the of animal-based ingredients was muscle meat of beef, poultry, deer, horse, lamb, rabbit, or goat, but also fish like coalfish, salmon, or trout was sometimes used. About 30 % of the animal-based products consisted of offal like rumen, psalter, kidney, spleen, udder, liver, lunge, chicken stomach, and heart. The remaining part depended on bones like chicken necks, wings, legs and carcasses, furthermore, different cartilages, ribs, spine, or veal bones.

Even when four owners did not feed any carbohydrates, in the mean 80 % of the plant-based products depended on vegetables and fruits, sometimes used in form of vegetable flakes

and about 20 % on carbohydrates like pasta, rice(-flakes), potatoes, barley, spelt, and oats.

All of the owners used different sorts of oil (sunflower, hempseed, walnut, black cumin, coconut oil, borage, olive, linseed, canola, safflower, primrose, salmon and cod liver oil).

In addition, seven of ten owners used dairy products, for example yoghurt, kefir, curd, buttermilk, and cottage cheese, as well as eggs.

In the mean owners used three supplements like egg shells, bone meal, brewer`s yeast, algae, garlic, healing earth, perna canaliculus, salt, propolis, milk thistle, artichoke and rose hip powder. Apart from individual components, some owners did also use herbal mixtures, different sorts of mineral feeds or BARF-minerals depending on plant components.
