# Supplementary material for: The fecal microbiome and metabolome differs between dogs fed Bones and Raw Food (BARF) diets and dogs fed commercial diets
Source: PLoS One. 2018 Aug 15;13(8):e0201279. doi: 10.1371/journal.pone.0201279 (PMC6093636; doi:10.1371/journal.pone.0201279)
Supplement: S2 File — (DOCX) [file pone.0201279.s004.docx]

**Studie zu gebarften Hunden**

Wir versichern, dass Ihre Daten selbstverständlich anonym behandelt werden und lediglich für Rückfragen bezüglich der Studie genutzt werden.

- **Allgemeine Daten zum Hund:**

- Name: __________________________

- Rasse: __________________________

- Alter: __________________________

- Geschlecht:

□ männlich □ unkastriert □ kastriert

□ weiblich □ unkastriert □ kastriert

- aktuelles Gewicht: _________________kg

- **Tiergesundheitsdaten:**

- Futteraufnahme: □ vermindert □ normal □ gesteigert

- Kotabsatz:

- Häufigkeit/ Tag: ________________________________________

- Konsistenz: ___________________________________________

- Farbe: _______________________________________________

- Blähungen: □ keine □ normal □ vermehrt

- Besonderheiten (Blut, Schleim, Pressen auf Kot,..):

_______________________________________________________________

- bisherige Erkrankungen:

- Art der Erkrankung(en): ____________________________________________

- Beginn der Erkrankung(en):_______________________________________

- **Fütterung:**

- Was bekommt das Tier zu fressen: ***jeweils bitte mit Mengenangaben in g pro***

***Tag/Woche***

- Fleischsorten:

□ Muskelfleisch, wenn ja, welches:

______________________________________________________________

______________________________________________________________

______________________________________________________________

_______________________________________________________________

□ Innereien, wenn ja, welche:

______________________________________________________________

______________________________________________________________

______________________________________________________________

□ Knochen, wenn ja, welche:

______________________________________________________________

______________________________________________________________

□ Schlundfleisch:

□ ja □ nein

Wo kaufen sie das Fleisch?

□ Metzgerei

□ verpackt, tiefgefroren: Firma: _________________________

_________________________

Name: _________________________

_________________________

Charge: ________________________

________________________

□ Sonstige: __________________________________________

Darreichungsform des Muskelfleisches/ der Innereien:

□ gewolft □ kleingestückelt □ ganze Teile

- Gemüse, Obst: ____________________________________________________________________

____________________________________________________________________

- Kohlenhydrate (z.B. Nudeln, Kartoffeln, Reis,...):

____________________________________________________________________

- Öl (z.B. Lein-/Distel-/Sonnenblumenöl): ____________________________________________________________________

- weitere Zutaten (z.B. Milchprodukte, Eier, Brot): ____________________________________________________________________

____________________________________________________________________

- Leckerlis: ____________________________________________________________________

- Supplemente, Futtermittelzusätze (z.B. Kräutermischung, Bierhefe): ____________________________________________________________________
