# Supplementary material for: The fecal microbiome and metabolome differs between dogs fed Bones and Raw Food (BARF) diets and dogs fed commercial diets
Source: PLoS One. 2018 Aug 15;13(8):e0201279. doi: 10.1371/journal.pone.0201279 (PMC6093636; doi:10.1371/journal.pone.0201279)
Supplement: S3 File — (DOCX) [file pone.0201279.s005.docx]

**Study on the subject of raw fed dogs (BARF)**

We assure you that your data will, of course, be treated anonymously and will only be used for inquiries regarding the study.

- **Characteristics of the dog:**

- Name: __________________________

- Breed: __________________________

- Age: __________________________

- Gender:

□ male □ un-neutered □ neutered

□ female □ un-neutered □ neutered

- Weight: _________________ kg

- **Health data of the dog:**

- Feed intake: □ reduced □ normal □ increased

- Defecation:

- Frequency/ day: ________________________________________

- Consistency: ________________________________________

- Colour: ________________________________________

- Flatulence: □ no □ normal □ increased

- Particularities (blood, mucus, pressing,..):

_______________________________________________________________

- Previous illnesses:

- Type(s) of disease: _________________________________________

- Beginning of disease: _________________________________________

- **Dog`s diet:**

- Contents of the feeding plan: ***in each case please with quantities (g per day/week)***

- Meat type:

□ Muscle meat, if so, which type of meat:

______________________________________________________________

______________________________________________________________

______________________________________________________________

_______________________________________________________________

□ Offal, if so, which type of offal:

______________________________________________________________

______________________________________________________________

______________________________________________________________

□ Bones, if so, which type of bones:

______________________________________________________________

______________________________________________________________

□ Maw meat:

□ yes □ no

Where do you buy the meat?

□ butcher`s shop

□ packed, frozen: Company _________________________

_________________________

Name: _________________________

_________________________

Batch-number: ________________________

________________________

□ others: __________________________________________

Type of crushing (meat/offal):

□ minced □ small pieced □ big pieces/whole parts

- Vegetables, fruits: ____________________________________________________________________

____________________________________________________________________

- Carbohydrates (pasta, potatoes, rice,...):

____________________________________________________________________

- Oil (linseed/thistle/sunflower oil,…): ____________________________________________________________________

- Further ingredients (milk products, eggs, bred, …): ____________________________________________________________________

____________________________________________________________________

- Treats: ____________________________________________________________________

- Supplements, feed additives (herbal mixtures, brewer`s yeast): ____________________________________________________________________
